# Plasma Levels of Matrilysins (MMP-7 and MMP-26) and Stromelysins (MMP-3 and MMP-10) in Diagnosis of Endometrial Cancer Patients

**DOI:** 10.3390/ijms26083824

**Published:** 2025-04-18

**Authors:** Ewa Gacuta, Michał Ławicki, Monika Kulesza, Paweł Ławicki, Aleksandra Kicman, Emilia Łubińska, Sławomir Ławicki

**Affiliations:** 1 Department of Gynecology and Gynecological Oncology, Medical University of Bialystok, M. Sklodowskiej-Curie 24a, 15-276 Bialystok, Poland; sunnyeve@wp.pl; 2 Department of Population Medicine and Lifestyle Diseases Prevention, Medical University of Bialystok, Waszyngtona 15B, 15-269 Bialystok, Poland; mlawicki@icloud.com (M.Ł.); monika.kulesza@sd.umb.edu.pl (M.K.); pawellawicki04@gmail.com (P.Ł.); doctorlubinska@gmail.com (E.Ł.); 3 Department of Aesthetic Medicine, Medical University of Bialystok, ul. Akademicka 3, 15-267 Bialystok, Poland; olakicman@gmail.com

**Keywords:** endometrial cancer, *adenocarcinoma endometrioides*, *myoma uteri*, MMP-7, MMP-26, MMP-3, MMP-10, CA125, matrilysins, stromelysins

## Abstract

Metalloproteinases (MMPs) play a significant role in cancer pathogenesis. We investigated the levels of MMP-7, MMP-26, MMP-3, and MMP-10 in comparison with the levels of a tumor marker (CA125) in the plasma of postmenopausal patients in early stages of endometrial cancer (EC) compared with control groups: patients with benign lesions (*myoma uteri*) and healthy controls. Plasma MMP levels were determined by ELISA and CA125 by CMIA methods. The study showed that plasma MMP-7 levels were significantly higher in EC patients compared to both control groups, whereas MMP-3 and MMP-26 levels were significantly higher in EC patients than in healthy women. MMP-7 showed the highest diagnostic sensitivity (SE), specificity (SP), positive (PPV) and negative predictive value (NPV), and diagnostic power (AUC) compared to other MMPs or CA125 in EC patients overall and patients with stage I and II EC. A combined analysis showed higher SE, NPV, and AUC levels in total EC patients and stage I and II EC patients—with the highest values for the combination MMP-7+CA125 (96%, 92%, 95%; 95%, 96%, and 98%; 0.9420, 0.9158, and 0.9693, respectively) or MMP-26 with CA125 (86%, 86%, and 86%; 59%, 73%, 73%; 0.8219, 0.8086, and 0.8353, respectively). The results suggest the usefulness of MMPs, especially MMP-7 and MMP-26, in combined panels with CA125 in diagnosing EC patients.

## 1. Introduction

Endometrial cancer (EC) is the most common malignancy of reproductive organs in Europe [1]. Most frequently, it occurs in postmenopausal women. However, it has been noted that its prevalence is shifting towards younger women as a direct result of the association between the etiopathology of EC and obesity. Besides advancing age and obesity, other EC risk factors include metabolic syndrome; increased estrogen exposure (hormone replacement therapy, early menarche, late menopause, polycystic ovary syndrome, chronic anovulation, lack of pregnancy, or tamoxifen therapy); or a genetic predisposition (Lynch syndrome or Cowden syndrome) [2]. Currently, there are no recommended screening tests for early EC detection in the general population. The initial diagnosis of EC involves taking a detailed medical history from the patient. Next, based on the symptoms, the diagnosis is expanded by a biopsy evaluation of endometrial tissue samples. When cancer is confirmed, imaging studies can additionally be used to assess the extent of the disease and the presence of possible metastases [3]. No circulating tumor markers with high sensitivity and specificity for EC are currently known. However, testing serum or plasma levels for CA125 in patients is often used for prognostic purposes and monitoring treatment [4,5,6,7].

Carcinogenesis is a complex process in which different phenomena occur simultaneously. These include an alteration of cell phenotype, a modification of the local immune response, a degradation of the extracellular matrix (ECM), and neovascularization. Metalloproteinases (MMPs) are enzymes capable of digesting almost all ECM macromolecules in tissues and are overexpressed in many cancers. MMPs are believed to be involved in every stage of carcinogenesis and play a particularly prominent role in invasion and metastasis [8]. The role of MMPs, especially MMP-7 and MMP-26, in the pathogenesis of endometrial cancer involves several related mechanisms. First, the activation of transcription factors, such as NF-κB, AP-1, and HIF-1α, is involved in cell proliferation and inhibition of cell apoptosis, which contributes to the growth of a tumor mass. Also associated with this process is an increase in cell sensitivity to growth factors—MMPs degrade ECM components that bind growth factors, which translates into an increase in their bioavailability and, consequently, an increase in cell proliferation. The degradation of the extracellular matrix is associated with ease of metastasis and alters cell–extracellular matrix interactions, which allows tumor cells to more easily migrate beyond the primary tumor. An equally important mechanism is the promotion of angiogenesis, which affects further tumor growth [8,9,10]. MMPs of the matrilysin (MMP-7 and MMP-26) and stromelysin (MMP-3 and MMP-10) groups also play an essential role in cancer pathogenesis [8,9]. MMP-7 not only has a broad substrate spectrum against ECM components (such as elastin, aggrecan, vitronectin, fibronectin, proteoglycans, and type IV collagen) [9] but also other non-ECM molecules, for example, MMP zymogens (proMMP-1, proMMP-7, proMMP-2, and proMMP-9); E-cadherin; Fas ligand; and syndecans [10]. MMP-7 is important in regulating certain processes, including aging, wound healing, and bone growth and remodeling; it is also a signaling pathway component that controls cell growth, inflammation, and angiogenesis [10,11]. MMP-3, MMP-10, and MMP-26 are expressed in normal tissues, such as the endometrium, bronchioles, genitourinary, and gastrointestinal tissues [8,9]. They can also function as oncogenic proteins, promoting tumor progression by inhibiting tumor cell apoptosis, reducing cell adhesion, and promoting angiogenesis [8,9,10]. Tissue overexpression or elevated blood levels of MMPs have already been observed in many malignancies [11], including common female cancers, such as breast cancer [12,13,14], ovarian cancer [15,16,17,18], cervical cancer [19,20,21], and EC [22,23,24,25].

Our project aimed to find a novel screening marker in material obtained by the most non-invasive possible method which does not directly interfere with the site of the ongoing neoplastic process. In this work, we present a continuation of the evaluation of matrilysins (MMP-7 and MMP-26) and stromelysins (MMP-3 and MMP-10) as novel tumor markers for women’s diseases [12,15,18]—this time, as screening biomarkers of EC.

## 2. Results

Plasma levels of tested parameters in EC patients and control groups are shown in Table 1. Total EC patients had significantly higher plasma concentrations of MMP-7 (median: 4.26 pg/mL), MMP-26 (median: 11.69 pg/mL), MMP-3 (10.31 pg/mL), and CA125 (median: 20.45 IU/mL) compared to healthy women (*p* < 0.0001, *p* < 0.0001, *p* = 0.004, and *p* = 0.027, respectively) or women with *myoma uteri* (only MMP-7; 2.82 pg/mL; *p* < 0.0001). Additionally, significantly higher concentrations of MMP-7 (2.82 pg/mL) were observed in the *myoma uteri* group in comparison to healthy women (*p* < 0.0001).

In stage I EC patients, significantly higher plasma levels of MMP-7 (median: 2.96 pg/mL), MMP-26 (median: 13.62 pg/mL), and MMP-3 (median: 10.10 pg/mL) were found in comparison to the group of healthy women (median: 1.23, 3.28, and 7.26, respectively). Similar statistically significant results were also found in stage II EC patients, MMP-7 (median: 4.93), MMP-26 (median: 8.77), and MMP-3 (median: 10.82), compared to healthy women (median: 1.23, 3.28, and 7.26, respectively). The plasma concentration of CA125 (22.70 U/mL) was significantly higher only in stage II EC patients in comparison to healthy controls (*p* = 0.002).

Additionally, significantly higher concentrations of MMP-7 and MMP-26 were also observed when comparing the *myoma uteri* group to the healthy controls (*p* < 0.0001 in both cases).

Furthermore, significantly higher plasma concentrations of MMP-7 and MMP-10 (916.3 pg/mL) were noted in stage II EC patients compared to the *myoma uteri* group (*p* < 0.0001; 0.041). Statistically higher plasma concentrations of MMP-7 were also observed when comparing patients with stage II EC.

We used a nonparametric test—Spearman’s rank correlation coefficient—to examine the correlations between MMP-7 and CA125 in the groups (Table 2). We found significant positive correlations between MMP-7 and CA125 in the total EC group (*r* = 0.20; *p* = 0.027) and between MMP-3 and CA125 in the healthy women group (*r* = 0.29; *p* = 0.041). Furthermore, we noted a significant positive correlation between MMP-7 and MMP-10 and a negative correlation with MMP-26 in the total EC group, as well as a positive correlation between MMP-3 or MMP-7 and MMP-26 in the *myoma uteri* group or between MMP-3 and MMP-7 in the healthy controls.

### Diagnostic Criteria of Studied MMPs and CA125

Table 3 presents the diagnostic criteria, sensitivity (SE), specificity (SP), and positive and negative predictive value (PPV and NPV), of the EC patients.

The highest SE for the total EC patients was found in MMP-7 (94%) in comparison to MMP-26 (78%) and other MMPs and CA125 (40%). The highest SP was observed for MMP-10 (94%), which was higher than the comparative marker CA125 (84%). An analysis of the combined parameters revealed that the highest SE was observed for MMP-7+CA125, up to 96% and the lowest SP in the combination of MMP-26+CA125 (38%).

Analyzing the subgroups, we noticed similar SE results with the total patients. In the stage I and II EC subgroups, the highest SE for a single parameter was found in MMP-7 (86% and 96%, respectively), which was higher than SE for MMP-26 (76% and 80%) and other MMPs or CA125 (34% and 46%, respectively). For the combined parameters, the highest increase in SE was noted in both subgroups for MMP-7+CA125 (92% for stage I EC and 96% for stage II EC), while SP was reduced in all combinations of MMPs with the tumor marker.

In the EC patient group, MMP-7 had a higher PPV (92%) and NPV (90%) than the comparative marker CA125 (83% and 41%, respectively) or other MMPs.

Analyzing the data of EC patients, we noted a higher PPV and NPV for MMP-7 in those with stage I EC (PPV: 86%; NPV: 92%) and stage II EC (88% and 96%, respectively), compared to the PPV and NPV of the comparative marker CA125 in the study groups (stage I EC: PPV: 68% and NPV: 56%; stage II EC: PPV: 74% and NPV 61%). Analyzing the parameter combinations, we found the highest increase in NPV for the total EC patients and in both subgroups for MMP-7 + CA125 (95%, 96%, and 98%, respectively) and a slight decrease in PPV for the total EC patients and in the subgroups for all combinations of matrilysins (MMP-7 and MMP-26) and stromelysins (MMP-3 and MMP-10) with CA125.

**Figure 1:**
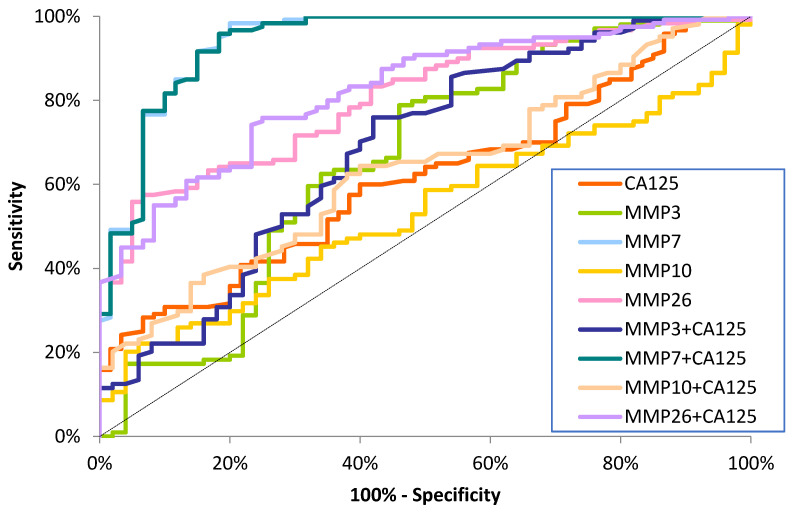
ROC curve for tested parameters in total EC patients.

**Figure 2:**
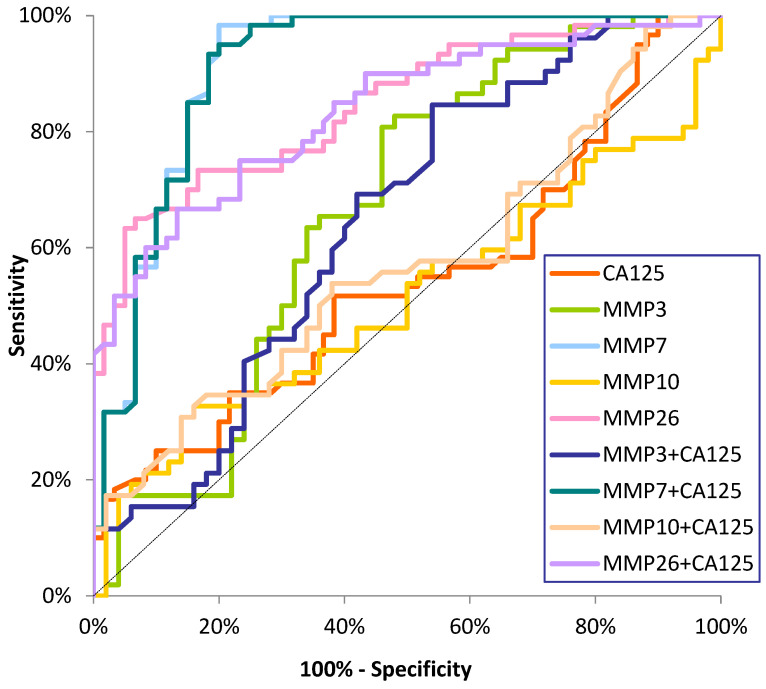
ROC curve for tested parameters in stage I EC patients.

**Figure 3:**
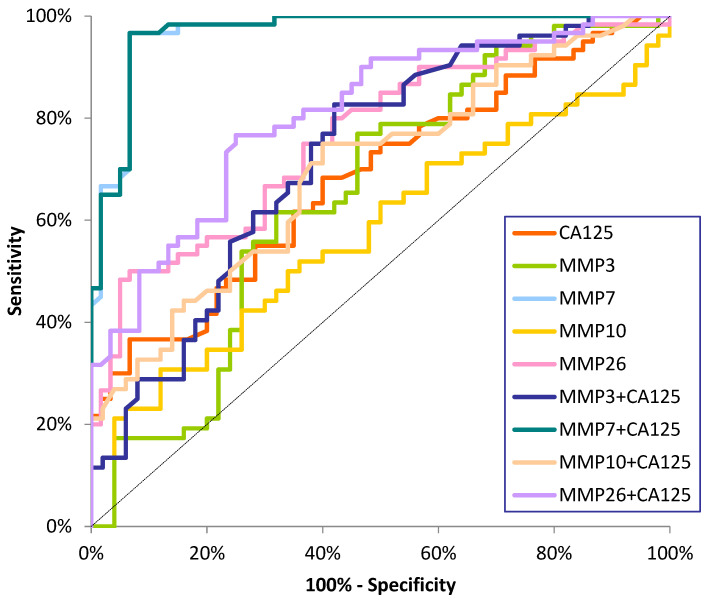
ROC curve for tested parameters in stage II EC patients.

The ROC curve illustrates the dependence of SE on SP for the parameters under study. In contrast, the potential clinical utility of a parameter as a tumor marker is demonstrated by the AUC while determining its diagnostic power. The detailed parameters of the ROC curve analysis are shown in Table 3 and Figure 1, Figure 2 and Figure 3.

The AUC for MMP-7 (0.9410) in the total EC patient group was higher than the AUC for the other tested MMPs and the comparative marker CA125 (0.6129). A combined analysis of MMPs with CA125 showed an increase in AUC values for the combined parameters: MMP-7+CA125 (0.9420) and MMP-26+CA125 (0.8219) (Figure 1).

We observed statistically significant diagnostic power for MMP-3, MMP-7, and MMP-26 for stage I and II EC patients, rather than AUC = 0.5, and CA125 only for stage II cancer patients. In both subgroups of EC patients, the AUC for MMP-7 was highest (0.9121; 0.9689), but it was also higher than that for MMP-26 (0.8458; 0.8542) and the other MMPs tested. The AUC for MMP-7 was also higher for CA125 (0.5428; 0.6831) and increased with tumor stages. In the analysis of combined matrilysins (MMP-7 and MMP-26) and stromelysins (MMP-3 and MMP-10) with the comparative marker, an increase in AUC was observed in stage I and II patients, with the highest values for MMP-7+CA125 (0.9158; 0.9693) (Figure 2 and Figure 3).

## 3. Discussion

Endometrial cancer is currently one of the most common cancers in women in Europe [1]. Despite the increasing number of EC cases, there are currently no recommended screening tests for its early detection, and markers, such as CA125 and HE4, are only used for prognosis and patient monitoring [4,5,6,7]. Therefore, it is necessary to search for additional laboratory methods for the early diagnosis of EC. The present work focuses on determining the screening utility of metalloproteinases, namely, matrilysins (MMP-7 and MMP-26) and stromelysins (MMP-3 and MMP-10), as modern tumor markers in patients with EC compared to women with benign lesions (myometrium) and healthy controls in independent analyses and in correlation with the routine marker CA125. At present, the number of studies on the screening utility of the studied MMPs in EC patients is scarce, so some of our results should be compared with those concerning other types of cancer.

MMP-7 and MMP-26 are usually expressed in healthy female endometria [26,27,28] and in various uterine lesions, such as endometriosis and myomas [26,29]. The expression of matrilysins is also found in patients with EC [23,30], where high levels of these MMPs have been associated with the presence of lymph node metastasis and a more advanced stage of the disease, leading to a poorer prognosis for patients [24,31]. These data indicate that MMP-7 or MMP-26 are involved in the progression of EC [24,32].

In our study, MMP-3, MMP-7, and MMP-26 levels were statistically higher in patients with EC than in healthy women. Guo et al. [21] reported similar findings and also noted higher levels of MMP-7 in patients with EC compared to healthy women. However, it should be noted that their study was conducted on serum. Importantly, in our study, patients with EC had higher plasma MMP-7 and MMP-10 levels than patients with benign lesions (*myoma uteri*), which may tentatively indicate the potential of using only MMP-7 as an auxiliary parameter in differentiating malignant from benign lesions or healthy controls (except for patients in stage I compared to those with myoma uteri) [21,32,33]. Guo et al. [21] observed higher levels of MMP-7 in women with EC compared to patients with benign lesions. Still, it is worth noting that their group consisted of patients with endometrial polyps and not *myoma uteri*. However, both endometrial polyps and *myoma uteri* are the most common benign tumors among women [27]. According to our knowledge, there are no other studies on MMP-7 or MMP-26 concentrations in plasma or serum of EC patients. There are, however, studies by Będkowska et al. [15] on ovarian cancer and Zhu et al. [19] on cervical cancer. Piskór et al. [12] also found higher MMP concentrations in cancer patients compared to healthy controls.

According to the literature, few reports concern the expression of MMP-3 and MM-10 in EC cells [31,34,35]. Unfortunately, our study did not yield statistically significant analyses of tested MMP-3 and MMP-10; however, our work focuses only on the early stages of cancer. We are interested in observing those initial stages where the lesion is minimally visible and beginning to develop.

Statistical correlations of MMP-7 were found in the EC groups. Moreover, concentrations were significantly higher in stage II EC patients compared to women at less advanced stages. This contradicts the findings reported by Gershtein et al. [22], who noted no correlation between MMP-7 concentrations and EC stages. In addition, Gershtein et al. [22] also found no correlation between MMP-7 concentrations and the degree of tumor spreading. No relationship between cancer stages and MMP-7 concentrations was found by Będkowska et al. [15] in ovarian cancer patients and Zhu et al. [19] in cervical cancer patients. In the case of breast cancer, there is a correlation between MMP-7 levels and cancer stages—higher levels of the marker are observed in stages III and IV compared to stage I [12]. Due to conflicting data, a clear determination of the relationship of MMP-7 concentrations to the early stages of EC will require additional studies. To the best of our knowledge, we are the first team to investigate the relationship between MMP-7 concentrations in the early stages of EC and healthy women and patients with *myoma uteri*.

In this study, diagnostic usefulness was assessed by indicators, such as sensitivity (SE) and specificity (SP) and positive (PPV) and negative (NPV) predictive values [36]. In our study, SE, SP, PPV, and NPV for MMP-7 and MMP-26 were higher than those of the mentioned diagnostic parameters for the routine marker CA125. A combined analysis of the tested MMPs, especially MMP-7 or MMP-26, with the comparative marker CA125 revealed an increase in SE and NPV. We observed minimal reductions in PPV and SP in the combined analysis of MMPs with CA125. We are the first research team performing SE, SP, PPV, and NPV analyses for matrilysins (MMP-7 and MMP-26) and stromelysins (MMP-3 and MMP-10) MMPs in patients with EC. At the same time, there are only a few studies evaluating tested MMPs in patients with other types of cancer. For cervical cancer, SE for MMP-7 was lower than that for the routinely used marker SCC-Ag, while SP was higher than SCC-Ag. Performing a combined analysis of MMP-7 and SCC-Ag, Zhu et al. [19] observed an increase in SE, while there was no increase in SP. Unfortunately, they did not analyze PPV and NPV. Similar analyses for MMP-7 and CA125 were conducted by Będkowska et al. [15] and for MMP-7 and MMP-26 in breast cancer patients [12].

In this study, regardless of the stage of EC, MMP-7 and MMP-26 values were more promising than those for CA125. This indicates that MMP-7 or MMP-26 could potentially be used to diagnose women at an early stage of EC. Earlier diagnosis of this cancer would translate into a better prognosis for patients due to earlier initiation of treatment [33].

Our study showed that the AUCs for MMP-3, MMP-7, and MMP-26 were statistically higher than AUC = 0.5 in total EC patients and patients at stages I and II compared to those for the prevalent marker CA125. Regardless of the group, MMP-7 and MMP-26 demonstrated higher diagnostic power than CA125, indicating the best diagnostic power of the tested matrilysins compared to the routine marker. We are the first team to determine the ROC curve in patients with EC, so our results cannot be compared to other findings. However, other research teams have also found higher AUCs for MMP-7 in other tumor types compared to other routinely used markers (e.g., CA125 or CA15-3) [12,15]. Notably, a combined analysis of the MMPs with CA125 resulted in an increase in AUC. This indicates the potential use of MMPs, especially MMP-7, in the early detection of EC and suggests the possibility of combining markers in more advanced stages of EC. However, this requires further research, especially given Będkowska et al. [15], who studied ovarian cancer, and Piskór et al. [12], who studied breast cancer, showed increased AUCs after combined analyses of MMP-7 with other parameters.

Unfortunately, this study has some limitations. First, as mentioned earlier, we did not perform analyses to determine the relationship between the MMP levels and tumor characteristics, such as size or stage. In addition, our study included only women with stage I and II EC. Despite these limitations, our study is novel; this article thoroughly demonstrates the potential screening utility of MMP-7 and MMP-26 in diagnosing women with early stages of EC, especially when combined with CA125.

## 4. Materials and Methods

### 4.1. Patient Eligibility

We included 120 patients with endometrial cancer (EC) who underwent diagnosis and subsequent surgical treatment in 2021–2023 at the University Clinical Hospital in Bialystok (Poland). Histopathological evaluations of EC were performed at the hospital diagnosis stage using preoperative endometrial tumor biopsy or intraoperative biopsy specimens. Patients were divided into groups by the stage of EC (I and II), according to 2009 FIGO criteria.

The characteristics of the study groups are shown in Table 4. All participants were postmenopausal women.

Our study involved patients with EC or a benign lesion (*myoma uteri*) diagnosed during gynecological examinations, followed by confirmatory examinations by an oncologist using imaging studies, including ultrasound (US)/magnetic resonance imaging (MRI), and laboratory tests. The selection of the study and control groups, as well as the preoperative therapeutic management, was carried out by the hospital unit following current clinical practice guidelines for the treatment of EC. Patients with malignant lesions who received adjuvant preoperative treatment were excluded from the study.

The healthy controls were volunteers who qualified for the study after an exam by a family doctor and then a gynecologist of the University Clinical Hospital in Bialystok, as well as participants of the Bialystok PLUS cohort study, on whom a detailed imaging diagnosis (abdominal or intravaginal US/MRI) and evaluation of laboratory results were performed, based on which the gynecologist subsequently determined their eligibility to participate in the study.

### 4.2. Biochemical Analysis

The study material was plasma from venous blood collected for the anticoagulant—lithium heparin. Venous blood was collected from participants and centrifuged at 1810× *g* for 10 min. The centrifuged plasma was then separated and stored at −81 °C until the day of the study.

We measured plasma concentrations of matrilysins (MMP-7 and MMP-26) and stromelysins (MMP-3 and MMP-10) using an enzyme-linked immunosorbent assay (ELISA) (Quantikine ELISA Human, R&D Systems Inc., Minneapolis, MN, USA). The assays were performed according to the manufacturer’s instructions provided with the kits, using double sample determinations for the standard curve and tested samples. To measure CA125 levels, we used a chemiluminescent microparticle immunoassay (CMIA) (Abbott, Chicago, IL, USA), according to the manufacturer’s protocols.

### 4.3. Statistical Analysis

The parameter analysis was performed using IBM SPSS Statistics for Windows, version 29.0.2.0 (Armonk, NY, USA: IBM Corp.).

After evaluating the normality of the distribution of the parameters with the Shapiro–Wilk test with Lilliefors correction, which revealed significant deviations from the normal distribution, we performed a statistical analysis using nonparametric tests. To assess statistical differences between two independent groups, we used the Mann–Whitney U test, whereas when comparing multiple groups, we used the Mann–Whitney U test with Holm–Bonferroni correction for multiple inquiries.

Medcalc’s Free Statistical Calculators were used to evaluate the diagnostic features of the single parameters, sensitivity (SE), specificity (SP), positive predictive value (PPV), and negative predictive value (NPV), and in the combined analysis of MMPs with CA125. The diagnostic power was analyzed using the area under the ROC curve (AUC), and the optimal cut-off points were determined by the 95th percentile of the healthy control group. Using the ROC curve, an analysis of the diagnostic reliability and diagnostic power of the tests was performed, with the optimal cut-off point determined for MMP-7 (2025 pg/m), MMP-26 (8.23 ng/mL), MMP-3 (7575 pg/mL), MMP-10 (2120,545 pg/mL), and CA125 (30.5 U/mL). Comparisons of ROC curves were made using AUCs determined by the nonparametric Youden method [37].

## 5. Conclusions

Accordingly, our results suggest the potential usefulness of the tested metalloproteinases, specifically, the matrilysins MMP-7 and MMP-26, in a combined panel with the comparative marker CA125 in diagnosing patients with EC.

## Figures and Tables

**Table 1:** Plasma levels of tested MMPs and CA125 in EC patients and control groups.

Tested Parameters		Endometrial Cancer (EC)			Control Groups	
		Total EC *n* = 120	Stage I *n* = 60	Stage II *n* = 60	*Myoma Uteri* *n* = 60	Healthy Women *n* = 60
MMP-7 (pg/mL)		*^1, 2^	*^1^	*^1, 2, 4^	*^3^	
	Median	4.26	2.96	4.93	2.82	1.23
	IQR	2.89–5.56	2.5–4.54	4.03–6.48	2.33–3.84	0.68–2.01
	Range	1.92–22.55	1.98–7.74	1.92–22.55	1.66–28.14	0.04–5.23
MMP-26 (pg/mL)		*^1^	*^1^	*^1^	*^3^	
	Median	11.69	13.62	8.77	15.98	3.28
	IQR	5.05–19.94	6.03–23.02	4.38–15.86	4.47–36.71	1.91–6.695
	Range	0.26–104.82	0.71–104.82	0.26–65.78	0.59–129.02	0.42–17.62
MMP-3 (pg/mL)		*^1^	*^1^	*^1^		
	Median	10.31	10.10	10.82	8.73	7.26
	IQR	8.08–13.60	8.13–13.57	7.69–14.08	5.93–13.37	4.97–11.81
	Range	2.02–41.29	4.08–41.29	2.02–29.75	2.34–22.61	1.81–44.80
MMP-10 (pg/mL)				*^2^		
	Median	789.52	731.6	916.3	654.73	725.22
	IQR	349.69–1606.61	265.86–1408.0	481.68–1659.74	330.0–870.40	454.76–1202.03
	Range	30.2–6665.42	69.6–4512.6	30.2–6665.4	20.56–9421.2	65.37–3006.1
CA125 (IU/mL)		*^1^		*^1^		
	Median	20.45	19.30	22.70	20.55	17.55
	IQR	14.2–29.85	11.46–27.66	17.0–33.5	13.33–29.73	12.88–23.70
	Range	7.25–374.5	8.04–358.9	7.25–374.5	3.94–154.1	4.24–39.94

*^1^ Statistical significance when comparing the cancer group and healthy controls. *^2^ Statistical significance when comparing the cancer group and the *myoma uteri* group. *^3^ Statistical significance when comparing the *myoma uteri* group and healthy controls. *^4^ Statistical significance when comparing groups with cancer at stages II and I. IQR—interquartile range; range—min.–max. range.

**Table 2:** Correlations between the levels of tested MMPs and CA125 in the total EC group, the *myoma uteri* group, and healthy women.

Group			CA125	MMP-3	MMP-7	MMP-10	MMP-26
Endometrial Cancer (EC)	CA125	*r*	1.00	0.07	0.20	0.19	−0.04
		*p* **		0.499	0.027	0.059	0.640
	MMP-3	*r*		1.00	0.03	0.17	0.09
		*p* **			0.738	0.080	0.380
	MMP-7	*r*			1.00	0.23	−0.18
		*p* **				0.017	0.049
	MMP-10	*r*				1.00	0.08
		*p* **					0.414
	MMP-26	*r*					1.00
		*p* **					
*Myoma Uteri*	CA125	*r*	1.00	0.15	0.08	−0.11	−0.02
		*p* **		0.283	0.527	0.439	0.891
	MMP-3	*r*		1.00	0.01	0.17	0.29
		*p* **			0.964	0.233	0.038
	MMP-7	*r*			1.00	0.02	0.35
		*p* **				0.876	0.006
	MMP-10	*r*				1.00	0.13
		*p* **					0.366
	MMP-26	*r*					1.00
		*p* **					
Healthy Women	CA125	*r*	1.00	0.29	0.23	0.07	0.12
		*p* **		0.041	0.072	0.628	0.363
	MMP-3	*r*		1.00	0.47	0.09	−0.04
		*p* **			0.001	0.553	0.791
	MMP-7	*r*			1.00	0.09	0.05
		*p* **				0.512	0.719
	MMP-10	*r*				1.00	0.08
		*p* **					0.590
	MMP-26	*r*					1.00
		*p* **					

Red—a statistically significant correlation. ** *p*-value is 0.001.

**Table 3:** Diagnostic criteria of tested parameters and characteristics of ROC curve in total EC group vs. controls.

Tested Parameter	Diagnostic Criteria	Endometrial Cancer (EC)		
		Stage I	Stage II	Total
MMP-7	SE (%)	86	96	94
	SP (%)	84	84	84
	PPV (%)	86	88	92
	NPV (%)	92	96	90
	AUC	0.9121	0.9689	0.9410
	SE_AUC_	0.0275	0.0143	0.0199
	95% CI	0.861–0.969	0.941–0.997	0.903–0.981
	*p* (AUC = 0.5)	<0.0001	<0.0001	<0.0001
MMP-26	SE (%)	76	80	78
	SP (%)	46	46	46
	PPV (%)	60	58	74
	NPV (%)	70	66	51
	AUC	0.8458	0.8542	0.8050
	SE_AUC_	0.0351	0.0431	0.0322
	95% CI	0.777–0.915	0.680–0.849	0.742–0.868
	*p* (AUC = 0.5)	<0.0001	<0.0001	<0.0001
MMP-3	SE (%)	76	80	78
	SP (%)	54	54	54
	PPV (%)	63	62	77
	NPV (%)	73	69	55
	AUC	0.6650	0.6542	0.6596
	SE_AUC_	0.0552	0.0553	0.0508
	95% CI	0.557–0.773	0.564–0.783	0.560–0.759
	*p* (AUC = 0.5)	0.0028	0.0053	0.0017
MMP-10	SE (%)	20	24	22
	SP (%)	94	94	94
	PPV (%)	77	80	88
	NPV (%)	54	55	38
	AUC	0.5112	0.5804	0.5346
	SE_AUC_	0.0585	0.0572	0.0469
	95% CI	0.396–0.626	0.468–0.692	0.443–0.627
	*p* (AUC = 0.5)	0.8489	0.1598	0.4603
CA125	SE (%)	34	46	40
	SP (%)	84	84	84
	PPV (%)	68	74	83
	NPV (%)	56	61	41
	AUC	0.5428	0.6831	0.6129
	SE_AUC_	0.0535	0.0484	0.0199
	95% CI	0.438–0.648	0.588–0.778	0.9031–0.9811
	*p* (AUC = 0.5)	0.4241	0.0002	0.0080
MMP-3+CA125	SE (%)	84	86	85
	SP (%)	48	48	48
	PPV (%)	62	62	77
	NPV (%)	75	77	62
	AUC	0.6460	0.7260	0.6860
	SE_AUC_	0.0552	0.0502	0.0478
	95% CI	0.538–0.754	0.627–0.824	0.592–0.780
	*p* (AUC = 0.5)	0.0082	<0.0001	<0.0001
MMP-7+CA125	SE (%)	92	96	96
	SP (%)	68	68	68
	PPV (%)	75	75	86
	NPV (%)	96	98	95
	AUC	0.9158	0.9693	0.9420
	SE_AUC_	0.0277	0.0142	0.0199
	95% CI	0.859–0.967	0.942–0.997	0.902–0.980
	*p* (AUC = 0.5)	<0.0001	<0.0001	<0.0001
MMP-10+CA125	SE (%)	44	56	50
	SP (%)	78	78	78
	PPV (%)	67	72	82
	NPV (%)	58	64	44
	AUC	0.5702	0.5702	0.6345
	SE_AUC_	0.0574	0.0574	0.0459
	95% CI	0.458–0.683	0.458–0.683	0.545–0.724
	*p* (AUC = 0.5)	0.2210	0.2210	0.0034
MMP-26+CA125	SE(%)	86	86	86
	SP (%)	38	38	38
	PPV (%)	58	58	74
	NPV (%)	73	73	58
	AUC	0.8086	0.8353	0.8219
	SE_AUC_	0.0363	0.0363	0.0311
	95% CI	0.764–0.906	0.764–0.906	0.761–0.883
	*p* (AUC = 0.5)	<0.0001	<0.0001	<0.0001

Red—a statistically significant correlation.

**Table 4:** The characteristics of the study groups.

Study Group	Group Size	Age (Median) Range (Min.–Max.)
Preoperative EC Total:	120	59 (51–71)
Stage I	60	58 (51–71)
Stage II	60	61 (58–70)
Benign Endometrial Lesions (*Myoma Uteri*)	60	56 (49–70)
Healthy Women	60	55 (48–69)

## Data Availability

The data presented in this paper are available upon request from the author due to the privacy of sensitive patient data.
